# Adjunctive Use of Platelet-Derived Concentrates (Platelet-Rich Plasma, Platelet-Rich Fibrin, Concentrated Growth Factor, Platelet-Poor Plasma) in Non-Surgical Periodontal Therapy: Current Evidence and Comparative Analysis

**DOI:** 10.3390/jcm15020554

**Published:** 2026-01-09

**Authors:** Sebastian Gawlak-Socka, Kinga Jeżewska, Natalia Bielecka-Kowalska, Sebastian Kłosek

**Affiliations:** 1 Student Scientific Association at the Department of Maxillofacial Surgery, Medical University of Lodz, Zeromskiego 113, 90-549 Lodz, Poland; sebastian.gawlak-socka@stud.umed.lodz.pl; 2 Student Scientific Association at the Department of Periodontology and Oral Mucosal Disease, Medical University of Lodz, Pomorska 251, 92-213 Lodz, Poland; kinga.jezewska@stud.umed.lodz.pl; 3 Department of Periodontology and Oral Mucosal Disease, Medical University of Lodz, Pomorska 251, 92-213 Lodz, Poland; sebastian.klosek@umed.lodz.pl; 4 Department of Oral Pathology, Medical University of Lodz, Pomorska 251, 92-213 Lodz, Poland

**Keywords:** periodontitis, platelet-rich plasma, platelet-rich fibrin, concentrated growth factor, platelet-poor plasma

## Abstract

**Background**: Periodontitis is a multifactorial, chronic inflammatory disease that leads to progressive destruction of the periodontal apparatus. Despite the effectiveness of scaling and root planing (SRP), residual inflammation and limited regenerative potential justify the search for adjunctive biologic therapies. Platelet-derived concentrates, including platelet-rich plasma (PRP), platelet-rich fibrin (PRF), concentrated growth factors (CGF), and platelet-poor plasma (PPP), have gained attention as autologous sources of growth factors enhancing periodontal regeneration. **Aim**: This narrative review provides a comparative analysis of the biological mechanisms, preparation protocols, and clinical outcomes associated with the adjunctive use of platelet-derived concentrates in non-surgical periodontal therapy. **Methods**: A narrative literature review was conducted using English-language publications retrieved from PubMed and Google Scholar, covering studies published from 2012 onward. The search strategy was based on combinations of keywords related to platelet-derived concentrates and non-surgical periodontal therapy. In vitro, in vivo, and clinical studies, as well as relevant narrative, systematic, and umbrella reviews evaluating the adjunctive use of platelet-derived concentrates (PRP, PRF, CGF, and PPP) were considered. Studies focusing on biological mechanisms, preparation protocols, and clinical periodontal outcomes were included, whereas case reports, studies unrelated to periodontal therapy, and publications lacking relevant clinical or biological outcome data were excluded. **Results**: Most clinical studies reported improvements in probing depth reduction, clinical attachment level gain, and bleeding indices following adjunctive use of platelet-derived concentrates with SRP. PRF tended to demonstrate more consistent clinical outcomes compared to PRP, potentially related to its simplified preparation and sustained release of bioactive molecules. CGF showed promising osteogenic and angiogenic properties in preclinical and early clinical studies. PPP, although less extensively investigated, exhibited regenerative and antimicrobial potential in preliminary reports. **Conclusions**: Platelet-derived concentrates may serve as valuable adjuncts in non-surgical periodontal therapy; however, the current evidence is characterized by methodological heterogeneity and variable study quality. While PRF appears to yield more consistent clinical results, definitive conclusions regarding superiority among different platelet concentrates cannot be drawn. Further well-designed randomized controlled trials are required, particularly for CGF and PPP.

## 1. Introduction

Periodontitis is a disease that has shown a marked increase in prevalence over the past several decades. Estimates from 1990 to 2010 indicated a considerably lower frequency of occurrence compared to estimates from 2011 to 2020, during which the prevalence of periodontitis among dentate individuals was reported at 62%, with severe periodontitis accounting for 23.6% [1]. Due to its high prevalence, periodontitis is often classified as a public health disease [1]. Data from studies conducted in the United States between 2009 and 2014 documented a prevalence of 42% among adults (30 years of age or older) affected by this condition [2]. Furthermore, evidence suggests a tendency toward improvement in population oral health, with a reduction in the incidence of severe periodontitis by approximately 2–5% among middle-income populations in several regions between 1990 and 2017. Conversely, in certain high-income countries, an increase in the prevalence of severe periodontitis by 1–3% among affluent individuals has been observed over the same period [3].

Periodontitis poses a threat to human health not only due to its high prevalence but also because of its pathomechanism and clinical consequences. The disease is characterized by progressive loss of alveolar bone and periodontal ligament, which, owing to its inflammatory nature, leads to irreversible destruction of the tooth-supporting structures. This process results in root exposure, increased tooth mobility, and ultimately tooth loss [4]. Periodontitis involves complex and dynamic interactions with other systemic diseases, including Alzheimer’s disease and mild cognitive impairment [5], diabetes mellitus [6], cardiovascular diseases [7], and COVID-19 [8].

As inflammation represents the primary pathogenic driver, therapeutic strategies aimed at controlling the inflammatory burden are central to periodontal management.

Non-surgical periodontal therapy, primarily based on scaling and root planing (SRP), constitutes the cornerstone of periodontitis treatment. SRP is effective in reducing subgingival biofilm and improving clinical parameters; however, its regenerative potential is limited, and residual inflammation may persist, particularly in deep periodontal pockets or in patients with systemic risk factors [4,9]. These limitations have prompted growing interest in adjunctive biological approaches capable of enhancing tissue healing and modulating host response within a non-surgical framework. Among emerging biologically based adjuncts, platelet-derived concentrates—including PRP, PRF, CGF, PPP—have attracted considerable attention. These autologous preparations provide a reservoir of growth factors, cytokines, and cellular components that may promote angiogenesis, soft tissue healing, and bone regeneration, while simultaneously exerting anti-inflammatory and antimicrobial effects [10]. Their application as adjuncts to non-surgical periodontal therapy represents a promising strategy to overcome some of the inherent limitations of SRP alone, Despite increasing interest, the available evidence remains fragmented. Direct comparative clinical studies between different platelet concentrates are scarce, and the majority of clinical data focus predominantly on PRP and PRF. In contrast, CGF and PPP remain relatively underexplored, particularly in non-surgical periodontal settings. Furthermore, heterogeneity in preparation protocols, outcome measures, and study designs complicates the interpretation and comparison of published studies.

Therefore, the objective of the present narrative review is to critically summarize and compare the biological rationale, preparation protocols, and available preclinical and clinical evidence regarding the adjunctive use of PRP, PRF, CGF, and PPP in non-surgical periodontal therapy. Particular emphasis is placed on identifying emerging trends, existing knowledge gaps, and directions for future research.

## 2. Periodontitis—Etiopathology, Pathomechanism

**Figure 1:**
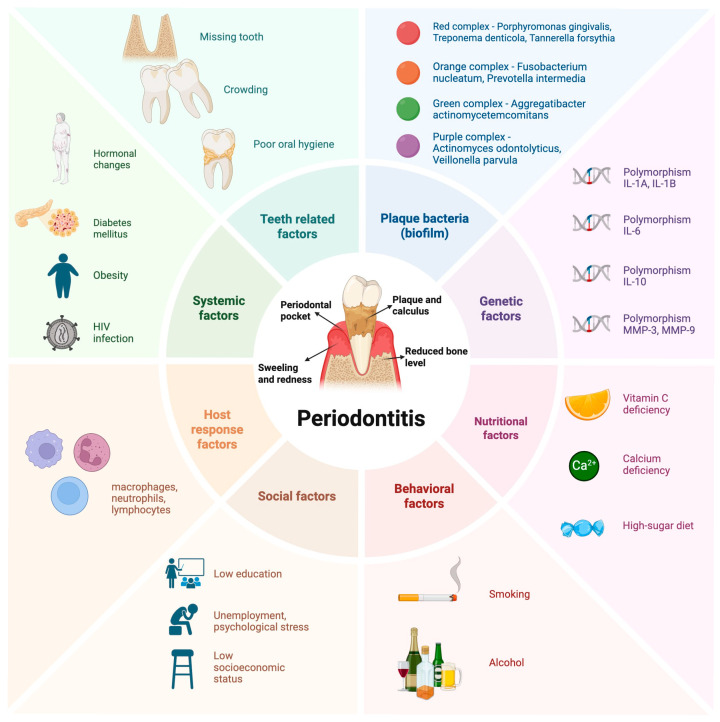
Etiopathogenesis of periodontitis. An illustration of periodontitis, resulting from the interplay of different factors.: teeth related, biofilm, genetic, nutritional, behavioral, social and systemic. The progression of the disease amplifies the host’s inflammatory reaction (involves host response factors, including macrophages, neutrophils, and lymphocytes), which contributes to increased pocket depth, connective tissue breakdown, and alveolar bone resorption. Created in biorender.com. Gawlak-Socka, S. (2025) https://BioRender.com/xpsp6vq (accessed on 5 December 2025) [16].

Periodontitis is a multifactorial inflammatory disease in which dental plaque, particularly its bacterial constituents, plays a pivotal role. Importantly, not all microorganisms present within dental plaque are directly associated with periodontal breakdown, as certain commensal species constitute part of the physiological oral microbiome [11]. The development of periodontitis depends not only on the presence of specific pathogens but also on the dynamics of biofilm formation, including the sequence of microbial colonization and the expression of virulence determinants [11]. In the early stages of biofilm development, pioneer bacteria, mainly streptococci, adhere to the acquired pellicle and initiate plaque formation. These initial colonizers modify the local microenvironment, facilitating the attachment of subsequent species, including the bridging organisms that connect early and late colonizers. As the biofilm matures, the reduction in oxygen availability promotes the selective growth of obligate anaerobes, many of which are closely associated with periodontal pathology [12]. The most common microorganisms implicated in the development of this condition belong to the so-called “red complex,” including *Porphyromonas gingivalis* and *Tannerella forsythia*, the “orange complex,” which includes *Fusobacterium nucleatum* and *Prevotella intermedia*, the “green complex,” represented by *Aggregatibacter actinomycetemcomitans* and the “purple complex”, which includes *Actinomyces odontolyticus* and *Veillonella parvula* [12,13]. These bacteria are primarily associated with the etiopathogenesis of periodontitis, as they are capable of effective colonization of the host and exhibit mechanisms that enable the evasion of host immune defenses [14]. It is important to emphasize, however, that microbial factors, although central to the onset of periodontitis, are not the sole contributors to its pathogenesis. Social, systemic, and genetic determinants, as well as local anatomical factors such as malposition and the crowding of teeth, also play a significant role (Figure 1) [15,16]. Additionally, tobacco smoking has been shown to exert a substantial impact on both the initiation and progression of the disease. Similar effects have been observed with the use of electronic cigarettes, due to the presence of nicotine [17,18].

The pathomechanism of periodontitis is as complex as its etiology and involves multiple inflammatory and immunological factors. The primary agents responsible for the disease are bacteria that colonize the cervical regions of teeth, forming dental plaque, also referred to as biofilm. This biofilm not only provides protection for microorganisms against antiseptics and antibiotics but also serves as a reservoir that supports their proliferation [4]. However, the mere presence of pathogenic microorganisms is not sufficient to induce disease. Additional contributing factors—such as poor oral hygiene, inadequate diet, and tobacco smoking—must also be present, as these disrupt the delicate balance between the resident oral microbiota and the host’s inflammatory response [19]. The progression of periodontitis is driven by a complex interplay between microbial factors and host immune responses. Local inflammation increases gingival crevicular fluid flow, providing nutrients that favor the proliferation of Gram-negative anaerobic bacteria, which subsequently trigger host defense mechanisms [20,21]. Bacterial lipopolysaccharides (LPS) activate the complement cascade, generating C3a and C5a fragments that stimulate the release of inflammatory mediators such as histamine, leukotriene B4, and prostaglandins. These mediators enhance vascular permeability and disrupt epithelial attachment, thereby facilitating tissue breakdown [20,21]. The activation of the adaptive immune system occurs through antigen presentation to T lymphocytes, which induces cytokine release and B cell activation [22]. Cytokines released during this process play a pivotal role in tissue destruction and alveolar bone resorption. Key pro-inflammatory cytokines such as IL-1β, IL-6, IL-17, and TNF-α, drive the progression of periodontal lesions, while cytokines such as IFN-γ, IL-4, IL-10, and IL-12 exert regulatory effects [23,24]. In addition, cytokine-induced expression of RANKL on osteoblasts and T helper cells stimulates osteoclast differentiation, resulting in alveolar bone loss [25,26]. Ultimately, periodontitis represents a dysregulated immune-inflammatory response, where cytokine-mediated interactions between immune cells disturb the balance between tissue protection and destruction [27].

These pathogenic mechanisms, characterized by persistent inflammation and impaired resolution, provide the biological rationale for the adjunctive use of plate-let-derived concentrates in non-surgical periodontal therapy. By delivering growth factors, cytokines, and fibrin matrices, platelet concentrates may modulate the host inflammatory response and support periodontal tissue healing and regeneration.

## 3. PRP (Platelet-Rich Plasma): Definition, Mechanism of Action, Composition, Preparation Method, and Application

Platelet-rich plasma is a first-generation platelet concentrate, used in regenerative medicine and dentistry for over 30 years. The term PRP was first mentioned by Kingsley in 1954. The PRP is a liquid form product of a two-step centrifugation of peripheral blood with anticoagulant agents [28]. Firstly, in tubes coated with ethylene diamine tetraacetic acid (EDTA), plasma is separated from red blood cells [29]. Subsequently, after the second centrifugation, an artificial coagulant is added to separate plasma from the platelets [29,30]. The platelet concentration is located above the baseline of the processed blood sample. However, the lack of standardization in the PRP preparation protocol hinders the analysis of research findings, as significant differences may occur between each PRP composition [28,30]. Depending on the technique used for PRP preparation, the required time varies from 30 to 60 min [29]. Platelet-rich plasma is composed of biologically active molecules that activate the healing cascade. The content of cytokines and growth factors is crucial for initiating the process. The variety of growth factors, such as platelet-derived growth factor (PDGF), transforming growth factor (TGF-β), and insulin-like growth factor (IGF-I), determines regenerative properties, thereby increasing efficiency of cell migration, proliferation, collagen synthesis, and osteoid formation. After PRP injection in the injured tissue, growth factors, cytokines, and lysosomes are released and initiate the healing process. At the same time, adhesion proteins start the hemostatic cascade, new connective tissue synthesis, and the neovascularisation process. Clinical studies have provided evidence supporting the regenerative potential of PRP [28]. Furthermore, antimicrobial properties of PRP to major periopathogens such as *Aggregatibacter actinomycetemcomitans* and *Porphyromonas gingivalis* were confirmed in in vitro studies. However, these findings have not yet been supported by robust clinical evidence. The clinical relevance of PRP’s antibacterial effects remains inconclusive [31]. The liquid form of PRP enables combination with bone biomaterials and forms “sticky bone” [32]. The application of PRP in soft tissue regeneration procedures, periodontal bone defect repair, and post-extraction sites has been documented in high-quality clinical studies [30]. Moreover, PRP can facilitate the non-surgical treatment of chronic periodontitis, serving as an adjuvant to scaling and root planing. It is applied to the bottom of the periodontal pockets after the professional mechanical plaque removal.

## 4. PRF (Platelet-Rich Fibrin): Difference from PRP, Duration of Action, Advantages in Periodontology

The platelet-rich fibrin is the second generation of platelet concentrates developed by Choukroun et al. [33]. The preparation protocol was simplified as the addition of any supplements is not required [32]. The anticoagulants included in the PRP preparation protocol were found to reduce the healing properties of the platelet concentrate as they interfere with platelet-mediated angiogenic and regenerative responses [32,34]. Depending on centrifugal time, speed, and tube type, different PRF types can be obtained [29]. Platelet-rich fibrin occurs in solid and liquid form (injectable PRF, i-PRF). The solid forms of PRF are leukocyte platelet-rich fibrin (L-PRF), titanium platelet-rich fibrin (T-PRF), and advanced platelet-rich fibrin (A-PRF). By decreasing the speed and duration of centrifugation, the i-PRF is produced, containing liquid thrombin and fibrinogen that has not yet converted to fibrin [32]. The heterogeneity of PRF formulation—including variations in leukocyte content, fibrin structure, and preparation protocols—affects their biological activity and clinical outcomes. Standardization of preparation methods is therefore critical to enhance the reliability and comparability of clinical evidence. On the other hand, the solid form is a dense clot with host platelets and leukocytes. The diversity of PRF forms enables its application in numerous clinical indications. In a collagen membrane form, PRF can be used in guided bone regeneration, recession coverage, intrabony defect repair procedures, and peri-implantitis [35]. While i-PRF can be applied with biomaterials during bone grafting, it improves the postoperative survival rate of diced cartilage and facilitates root coverage with free gingival graft surgery as sticky bone [29]. PRF promotes healing and regeneration by modulating the immune response, stimulating angiogenesis, traps circulating stem cells, and induces collagen type 1 synthesis. What is more, PRF stimulates the growth of fibroblasts and osteoblasts and suppresses osteoclastogenesis. The platelet-rich fibrin can reduce postoperative pain and lower the risk of wound infection due to its anti-inflammatory and antimicrobial properties [32,36]. According to Ozsagir et al.’s [30] investigation, a combination of micro-needling with i-PRF may increase gingival thickness in patients with thin phenotypes. Furthermore, i-PRF was applied with a positive outcome to standard SRP for patients with periodontitis. The clinical attachment level, gingival margin levels, pocket depths, and bleeding on probing were improved compared to SRP alone [9,33]. The healing properties of PRF seem to be more effective in soft tissue repair than when applied to hard tissue regeneration [33,35]. Comparative advantages of PRF over PRP—such as simplified preparation, lower cost, and the absence of exogenous additives—have been frequently reported [29,33]. In vitro studies, including the work of Wang et al. [37], have demonstrated higher levels of selected growth factors and extracellular matrix components in i-PRF compared to PRP; however, due to variability in preparation protocols and limited head-to-head randomized clinical trials, definitive conclusions regarding clinical superiority cannot yet be drawn [29].

## 5. CGF (Concentrated Growth Factor): Newer Generation, Advantages over PRF, Properties

In 2006, Sacco introduced the third generation of autologous platelet concentrate, concentrated growth factors [38]. CGF is obtained through centrifugation of peripheral blood using a variable-speed protocol, resulting in a fibrin matrix that is denser than that of PRF [34,39]. The preparation involves plastic tubes containing silica particles and allows for CGF to be obtained in both gel and liquid forms [38].

Modifications in centrifugation parameters enable the concentration of growth factors and stem cells within a relatively small plasma volume. Compared with PRP and PRF, CGF has been reported to exhibit slower degradation after application and a sustained release of growth factors for up to approximately 14 days; however, these characteristics are largely derived from in vitro and surgical regeneration studies [40].

Most available evidence regarding CGF focuses on bone regeneration, angiogenesis, and soft tissue healing in surgical contexts, including implantology and grafting procedures. Some studies have suggested favorable biological properties of CGF compared with PRF, particularly in terms of fibrin density and growth factor retention; nevertheless, direct comparisons remain limited and heterogeneous [34,38,41]. In periodontal bone regeneration, CGF and PRF have demonstrated largely comparable outcomes [34].

Importantly, clinical evidence supporting the use of CGF in non-surgical periodontal therapy is currently limited, and only a small number of studies have addressed this application directly. As a result, conclusions regarding superiority over PRF or PRP in non-surgical settings cannot be drawn at present—in the Li et al. [41] investigation, CGF presented more effective bone induction and tissue regeneration potential than both PRP and PRF, contrary to a study conducted by Lei [42], where A-PRF exhibited a larger amount of growth factors and a more sustainable release period. Further well-designed randomized controlled clinical trials are required to clarify the role of CGF as an adjunct to scaling and root planing and to determine its clinical relevance in periodontal therapy without surgical intervention.

## 6. PPP (Platelet-Poor Plasma): Current Evidence and Potential Biological Properties

After the blood sample is centrifuged, it is divided into three layers: platelet-poor plasma, platelet concentrate, and variable leukocyte and erythrocyte fraction. The PPP fraction was not considered therapeutically beneficial and discarded. However, in this layer, insulin-like growth factor-1 (IGF-1) and hepatocyte growth factor (HGF) are mainly present. As both reside outside of the platelet alpha granules, platelet concentrates contain lower levels of IGF-1 and HGF compared to PPP (Table 1) [43]. These growth factors induce angiogenesis, inhibit inflammation, and fibrosis. Moreover, the PPP promotes cell migration, bone and myofibroblastic differentiation, and actin remodeling [44]. The cell signaling is exerted by the exosomes and macrovesicles’ content. The obtaining of protein concentrate is possible due to newly developed ultrafiltration technologies. Plasma water, cytokines, molecules, and plasma proteins with molecular mass less than the pore size of fibers are eliminated. The PPP contains proteins such as fibrinogen, albumin, and alpha-2-macroglobulin. However, when the mechanical properties of PPP membranes were compared with those of A-PRF and CGF, it was found that PPP membranes were weaker and more degradable [45]. The platelet-poor plasma can be applied alone or in combination with PRP, forming protein-rich platelet concentrate (PR-PRP). The consolidated product initiates interactions between macrophages, fibroblasts, and mesenchymal stem cells and molecules and cells in PRP [42]. In a study by Song, the PPP was found to positively impact nerve regeneration in peri-implant bone [46]. In another investigation, Hatakeyama et al. [47] found that platelet-poor plasma was effective in the preservation of sockets with buccal dehiscence. In an in vitro study, the application of PPP was proven to induce gingival repair and osteoblastic differentiation of stem cells from periodontal ligament [39]. Furthermore, antimicrobial properties of PPP to major periopathogens such as *Aggregatibacter actinomycetemcomitans* and *Porphyromonas gingivalis* were confirmed [32]. While PPP exhibits promising characteristics, it is important to emphasize that these observations are primarily based on preclinical studies. The translation of such properties to clinical applications remains largely speculative. Therefore, further investigations are essential to generate robust evidence supporting the efficacy of PPP in in vivo regenerative procedures.

## 7. PRF in Non-Surgical Treatment

The role of PRF in non-surgical periodontal treatment was thoroughly investigated over the years. The solid form of PRF was used as a thin membrane and inserted in pieces into the periodontal pocket after SRP [48,49]. The presence of i-PRF facilitated its application and became an effective drug carrier for antibiotics such as metronidazole and ciprofloxacin. The combination of both platelet concentrate and antibiotic leads to greater improvement in periodontal parameters [50,51,52].

In Chandrasekar’s study [50], 10 participants with stage II-III periodontitis were divided into two groups. Clinical parameters (OHI, GI, BOP, PPD, CAL) were assessed at baseline, 4 weeks and 3 months after treatment. In the first group, patients received i-PRF alone after non-surgical periodontal treatment (SRP); in the second group, metronidazole-infused PRF gel was applied subgingivally with a sterile syringe after SRP. To prepare the gel, the PRF clot was transferred to a test tube containing 2 mL of 0.5% metronidazole. The gelation was facilitated with an incubation for 10 min at room temperature. When the process was completed, the gel product was inserted into periodontal pockets. Significant improvement in PPD and CAL in both groups occurred.

The metronidazole-infused PRF gel group demonstrated a more pronounced reduction in GI and BOP compared to the i-PRF group. In another study, L-PRF was combined with metronidazole, and the efficacy of L-PRF in reducing *Porphyromonas gingivalis* concentrations was evaluated. Gingivalis was measured at the baseline and 1 month after treatment. Clinical parameters (CAL, MGI, BI) were evaluated at baseline, 1, 3, and 6 months post-procedure.

The preparation of L-PRF loaded with metronidazole included dissolving 250 mg metronidazole tablets in 25 mL saline; 0.5 mL was added to the patient’s blood sample, centrifuged, and the received clot was squeezed out. Such a prepared membrane was applied subgingivally into the pockets using a plastic filling instrument. The Perio-pack material was applied to cover the operative field. In both groups, clinical parameters were improved, and *P. gingivalis* concentration was decreased. L-PRF with metronidazole showed superior results; however the difference was not statistically significant [52]. i-PRF role as a LDD vehicle for the delivery of ciprofloxacin in periodontal pocket therapy was investigated. A total of 79 periodontally diseased pockets sites were divided into three groups: group 1 (*n* = 25), SRP + i-PRF + Cip; group 2 (*n* = 25), SRP + i-PRF; group 3 (*n* = 25), SRP without any adjunctive intervention. Periodontal parameters (PD, CAL, GI, PI) and relative quantification of *A. actinomycetemcomitans* at the 12th week [51]. However, no significant difference in Porphyromonas gingivalis concentration was noticed between application of L-PRF + metronidazole and L-PRF alone [52]. In Sherif et al.’s study [53], the combination of i-PRF and vitamin C was applied to periodontal pockets as an adjunct to the SRP protocol. 45 patients with stage-II grade A periodontitis were separated into 3 groups. Clinical parameters (BOP, PD, CAL, GM, PI, and radiographic bone loss were assessed at baseline, 3 months, and 6 months post-treatment. Post-operative pain was evaluated on the second and third day post-treatment.

Firstly, full-mouth professional mechanical plaque removal (PMPR) was performed. In the PMPR + i-PRF/VitC group, 2500 µg of pure VitC was added to the blood sample in order to obtain a concentration of 250 µg/mL and centrifuged. The i-PRF was collected using the stent and the insulin syringe and applied for 15 min to the bottom of periodontal pockets [52]. Nevertheless, authors observed no significant improvement between control (PMPR alone) and test groups (PMPR + i-PRF, PMPR + i-PRF + vitamin C). On the other hand, the i-PRF’s ability to reduce pain was evaluated in the studies conducted by Sherif and Al-Rihaymee; both research teams confirmed i-PRF’s positive influence on postoperative pain decrease [48,53]. As periostin is a reliable marker for tissue regeneration, its concentration in gingival crevicular fluid after SRP with i-PRF application was measured. In the study, 14 patients with contralateral periodontal pockets with 4–6 mm in depth were included; sites were divided into the control group (SRP alone) and the test group (SRP + PRF). Periostin level in GCF and clinical periodontal parameters (PPD, CAL, PI, BOP) were measured at baseline, 1 month, and 3 months after the procedure. PRF turned out to have a significant impact on the periostin level, leading to its increase and healing enhancement [47]. What is more, the application of PRF led to both postoperative pain and treatment time reduction. The influence of i-PRF application on the concentration of TGF-β and collagen-1 (Col-1) in gingival crevicular fluid was investigated by Özcan E. et al. at periodontal para-baseline, third, seventh, and fourteenth days post-treatment. Significantly higher levels of TGF-β and Col-1 were found in the test group (SRP + i-PRF) than in the control group (SRP alone) [49]. The effectiveness of i-PRF as an adjunct to non-surgical periodontal treatment in patients with grade C periodontitis was evaluated. In Shunmuga’s study, patients with chronic periodontitis and type 2 diabetes were treated. Periodontal parameters (PPD, PI, MGI, CAL, BOP) were assessed at baseline, 3 months, and 6 months post procedure. The test group (SRP + i-PRF) results were not superior to the control group (SRP + saline) [54]. However, when i-PRF was applied to enhance the SRP result to smokers with periodontitis stage 2–3 and grade C, a great periodontal parameter improvement in comparison to the test group (SRP + saline) was observed (Table 2) [55].

Overall, available clinical studies suggest that the adjunctive use of PRF and i-PRF in non-surgical periodontal therapy may contribute to short-term improvements in selected clinical and biological parameters, including probing depth reduction, bleeding indices, pain reduction, and biomarkers of tissue regeneration. However, the evidence remains heterogeneous and inconsistent, with several studies reporting no significant superiority over SRP alone, particularly in patients with systemic conditions such as type 2 diabetes. Variations in PRF formulation, delivery protocols, adjunctive agents (e.g., antibiotics or vitamin C), and patient characteristics limit direct comparison between studies. Consequently, while PRF-based adjuncts appear promising, the overall strength of evidence is moderate to low, and conclusions should be interpreted cautiously.

## 8. PRP: Effectiveness, Comparison with PRF

The benefits of PRP application as an adjunct to the SRP protocol were confirmed in the Agarwal et al. study [56], where patients were divided into the test group and the control group. Participants in the test group were treated with SRP followed by subgingival PRP gel application, whereas patients in the control group received placebo gel after SRP. A significant CAL increase was observed in patients who underwent combined treatment [56]. Anti-inflammatory properties of PRP + SRP were assessed in a different study by the lymphocyte count analysis. The procedure protocol included supragingival and subgingival scaling, root planing with universal curettes, and PRP injection to the bottom of the pocket. Both on the day of the intervention and during follow-up visits, the number of lymphocytes in 1 mL of the patient’s blood was measured. One month post-procedure, a reduction in lymphocyte level was noticed [57]. Furthermore, i-PRF and PRP efficiency as adjuncts to SRP were compared. The study included 70 medically healthy adult patients, separated into three groups: PRP group, i-PRF group, and control group. Two bilateral interproximal defects of single-rooted teeth were selected in the same arch by the split-mouth technique. Following scaling and root planing, the test sides received i-PRF or PRP injected directly into the gingival sulcus with a microneedle. Repetition of the injection took place 14 and 28 days after. i-PRF had a greater positive influence on clinical parameters than PRP [58]. In the randomized split-mouth study conducted by Sharaki [58], the efficiency of PRP and Nd: YAG laser as adjuvants to SRP was compared. All sites received standard non-surgical mechanical debridement prior to adjunctive interventions. The PRP was applied immediately after SRP into periodontal pockets with a sterile syringe. On the laser side, an Nd: YAG laser was delivered into the periodontal pocket with a fiber tip (300 µm in diameter) inserted subgingivally. The inferiority of PRP to Nd: YAG was proven, as laser application resulted in greater improvement of periodontal parameters (Table 3) [9,59]. Although some studies reported more favorable short-term outcomes for PRF or i-PRF compared to PRP, direct comparisons are limited by heterogeneity in study design, preparation protocols, and outcome measures, and no definitive superiority of one adjunct over another can be established.

## 9. CGF: Number of Available Studies, Results

The concentrated growth factors occur in two forms: gel-phase (GPCGF) and liquid-phase (LPCGF). As gel-phase CGF application has been widely studied and has established its role in the hard tissue regeneration and surgical periodontal treatment, the utility of liquid-phase CGF is poorly investigated. However, a few promising in vitro studies are available. A study conducted by Niemczyk et al. [59] revealed that LPCGF can be successfully applied as a drug carrier for fluconazole and voriconazole against *Candida albicans*, *Candida glabrata*, and *Candida kruzei* [60]. The LPCGF maintains the composition of active molecules such as TGF-β and vascular endothelial growth factor (VEGF), as well as their biological activity, when compared to conventional CGF. The presence of CD34+ cells in LPCGF, together with its capacity for gradual release of growth factors, supports the long-term inhibition of inflammation-related mediators. Furthermore, in a different in vitro study, LPCGF was proven to effectively carry the combination of amoxicillin and metronidazole [61]. In an investigation conducted by Zahn, the biological effect of LPCGF with EDTA on periodontal teeth was assessed. The combined treatment was proven to significantly increase cell differentiation-related genes. What is more, the application of EDTA and/or LPCGF to the root enhances periodontal ligament cell proliferation and migration more than SRP [62]. Although the results of in vitro studies are promising, further research is still needed to confirm the use of LPCGF in the non-surgical treatment of periodontal diseases. Importantly, to date, no randomized clinical trials have evaluated liquid-phase CGF as an adjunct to non-surgical periodontal therapy in humans. Therefore, current evidence supports LPCGF primarily as a promising experimental biomaterial, and its potential clinical application in non-surgical periodontal therapy should be regarded as a future research direction rather than an established clinical biomaterial.

## 10. PPP: Is It Employed in Clinical Practice?

The role of PPP in regenerative medicine and dentistry is still being investigated. The plasma gel (PG) is a product of heating PPP, as high temperature causes denaturation and the formation of a gel-like substance. The Cheng et al. [63] in vivo investigation aimed to evaluate its absorption rate and regenerative efficiency on animal models. In this study, plasma gel was mixed with PRP and was injected subcutaneously into nude mice. The plasma gel was found to rapidly disappear; 50% of volume was absorbed in the first week, and after eight weeks, almost completely. Even though significant absorption was observed, a positive effect on neovascularisation and an increase in collagen deposition was noticed. Nakamura et al. [64] studied the plasma gel application as a drug carrier for controlled release. The study included polyphosphate, a substance considered a hemostatic agent and bone regeneration material with a short half-life in the human body. Plasma gel can serve as an effective carrier for polyphosphate in tissue engineering by trapping it through interactions with divalent cations, which stabilize the otherwise difficult-to-retain linear polyphosphate within the gel matrix. In another work, PG was used as a carrier of epigallocatechin-3-gallate (EGCG), an antioxidant compound. The PG was proven to be efficient as a drug carrier with sustainable release of EGCG [65]. Taken together, the plasma gel derived from platelet-poor plasma in the future may be used as an adjunct to non-surgical periodontal treatment, delivering drugs and promoting tissue regeneration via neovascularization and collagen synthesis. However, further investigations are needed to thoroughly determine the PPP application. Considering the lack of robust clinical trials, the application of PPP and CGF currently remains as a future perspective rather than a clinical recommendation.

## 11. Limitations

This review has several limitations that should be considered when interpreting the findings. First, as a narrative review, it does not follow the systematic methodology of a meta-analysis, and the selection of studies may be influenced by publication bias. Second, the included studies vary widely in study design, patient populations, follow-up duration, and platelet concentrate preparation protocols, which limits comparability. Third, the review integrates results from both human clinical studies and animal or in vitro experiments. While preclinical studies provide mechanistic insights, their findings cannot be directly extrapolated to clinical outcomes in humans. Finally, the heterogeneity and variable quality of the available evidence prevent definitive conclusions regarding the comparative efficacy of different platelet-derived concentrates. Some reviews suggest a trend toward more consistent clinical outcomes with PRF compared to PRP; however, these findings should be interpreted with caution due to the heterogeneity in study design, patient populations, and preparation protocols. Moreover, specific patient-related factors, such as the presence of systemic conditions (e.g., type 2 diabetes mellitus), may influence treatment response and limit the generalizability of reported results. Additionally, alternative adjunctive approaches, such as laser-assisted therapy, have demonstrated comparable or superior outcomes in selected clinical scenarios.

Importantly, the present work represents a narrative review, and the available body of evidence is characterized by considerable methodological heterogeneity, short follow-up periods (typically ranging from 1 to 6 months), and variable study quality. Therefore, current findings primarily reflect short-term clinical effects, and no definitive conclusions regarding long-term outcomes or the superiority of one platelet concentrate over another can be drawn.

Despite these limitations, the review provides a comprehensive overview of current knowledge on the adjunctive use of platelet concentrates in non-surgical periodontal therapy and highlights areas for future well-designed randomized controlled trials.

Future research should focus on well-designed, adequately powered randomized controlled trials with standardized preparation protocols, uniform outcome measures, and extended follow-up periods to better clarify the clinical relevance, durability of effects, and comparative effectiveness of different platelet-derived concentrates in non-surgical periodontal therapy.

## 12. Conclusions

The available evidence suggests that the adjunctive use of platelet-derived concentrates enhance short-term clinical outcomes of non-surgical periodontal therapy when scaling and root planing. While the clinical benefits of PRF and PRP have been more extensively investigated and appear more consistently reported, the evidence regarding PPP and CGF remains limited and inconclusive, particularly in non-surgical periodontal therapy. Further well-designed clinical trials are required to clarify the long-term comparative clinical value of different platelet-derived concentrates.

## Figures and Tables

**Table 1:** The differences between platelet concentrates.

	Form	Preparation Protocol	Platelet Concentration	Leukocytes	Growth Factor Release Rate
PRP	Liquid	Two-step centrifugation, lower speed to separate red blood cells higher speed to concentrate platelets	1.0–3.3 × 10^6^/µL	Depending on the preparation protocol	Average
					
					
PRF	Liquid or solid	Single centrifugation without artificial anticoagulant agents	L-PRF—0.4–0.9 × 10^6^/µL A-PRF—3–6 × 10^6^/µL	+	L-PRF—Slow A-PRF—highest
					
CGF	Gel	Single centrifugation with repeatedly switched speed	3.1–5.4 × 10^6^/µL	+	Average
					
					
PPP	Liquid	Obtained during PRP preparation	<0.01 × 10^6^/µL	-	Slow
					

The summary of the main features of blood-derived products, form, preparation protocol, content of platelets, and leukocytes, and the comparison of growth factors release rate [28,29,30,31,32,33,34,35,36,37,38,39,40,41,42,43,44,45,46]. PRP—platelet-rich plasma; PRF—platelet-rich fibrin; A-PRF—advance platelet-rich fibrin; L-PRF—leukocyte platelet-rich fibrin; CGF—concentrated growth factor; PPP—platelet-poor plasma.

**Table 2:** The review of available studies investigating the role of PRF in a non-surgical periodontal treatment.

Authors, Year, Reference, Journal, Study Design	Intervention	Sample Size	Follow-Up Period	Outcomes
Al-Rihaymee S. et al. [48] Journal of Cellular and Molecular Medicine, clinical trail	PRF application as adjuvant to SRP, evaluation of periostin level in gingival cervical fluid	14 patients, 14 control sites, 14 test sites (split mouth technique)	3 months	Periodontal parameters improvement, increased periostin level, healing enhancement, postoperative pain reduction and reduction in treatment time
				
				
Özcan E. et al. [49] Journal of Periodontology, A randomized controlled split—mouth clinical study	PRF applied after SRP, measurement of concentration of TGF-β and Col-1 in gingival crevicular fluid	12 patients, 24 periodontal pockets (split mouth technique)	6 months	Healing improvement, increase in Col-1 and TGF-β expression
				
Chandrasekar D. et al. [50], Journal of oral biology and craniofacial research, a clinical study	Application of i-PRF and metronidazole-infused i-PRF in non-surgical periodontal treatment	10 patients, 20 sites (split mouth technique)	3 months	Clinical periodontal parameters improvement
				
				
Thamaraiselvan M. et al. [51] Journal of Advanced Periodontology & Implant Dentistry, randomized controlled clinical trial	The ciprofloxacin [Cip])-loaded i-PRF as a LDD system adjunct to subgingival debridement	79 periodontal pocket sites	12 weeks	Improvements in clinical and microbial parameters assessed
				
Omar Y.K. et al. [52] BMC Oral Health, a randomized controlled clinical trial	L-PRF as a locally sustained release device for metronidazole antimicrobial	24 patients, with 80 periodontal pockets	6 months	clinical parameters were improved, and *P. gingivalis* concentration was decreased.
Sherif M.A. et al. [53] BMC Oral Health	i-PRF with vitamin C and i-PRF effect on professional mechanical plaque removal	45 patients	6 months	Positive outcome of i-PRF and i-PRF + vitamin C application on postoperative pain. Comparable improvement of clinical parameters in both groups
Shunmuga P.D. et al. [54], Clinical advances in periodontics, a randomized controlled trial	i-PRF as adjunct to SRP in patients with type 2 diabetes	26 patients	6 months	Both methods were effective in periodontal pockets treatment
Çağıran Gürbüz T. et al. [55] BMC Oral Health	The i-PRF as an adjunct in non-surgical periodontal treatment in smokers with periodontitis	25 patients	3 months	Significant PD and CAL reduction was observed in the group treated with SRP + i-PRF

The table summarizes studies evaluating PRF use in non-surgical periodontal therapy. SRP—scaling and root planing; PRF—platelet-rich fibrin; TGF-β—transforming growth factor; Col-1—collagen 1; i-PRF—injectable PRF; LDD—local drug delivery.

**Table 3:** The main studies (2012–2025) of PRP application in non-surgical periodontal treatment.

Reference	Application of PRP	Methodology	Evaluation Parameters	Follow-Up Period	Conclusion
[56] Agarwal A. et al. (2014)	adjunct to SRP protocol	87 non-smokers suffering from moderate to severe chronic periodontitis were selected and divided into 2 groups (SRP + placebo gel, SRP + PRP)	Periodontal parameters: PPD, mBI, PI, CAL	Baseline, 3 months, and 6 months after treatment	The significant increase in CAL in the test group
					
					
[57] Abdul Ameer L.A. et al. (2018)	adjunct to SRP protocol effect on the lymphocyte count	20 patients with chronic periodontitis and pockets depth ≥ 4 mm	The lymphocyte count and periodontal parameters (PD, GI, PI, BOP, CAL)	Baseline and 1 month post-procedure	PRP significantly reduced lymphocyte count and improved periodontal parameters, indicating an anti-inflammatory effect
					
[58] Amin A. et al. (2022)	The comparison of PRP and i-PRF as adjuvants to SRP	70 medically healthy adult subjects with chronic periodontitis, divided into 3 groups (SRP + PRP, SRP + i-PRF, SRP alone)	Periodontal parameters (PD and CAL)	Baseline, 1, 2, and 3 months post-procedure	i-PRF showed greater and more sustained improvement of periodontal parameters compared with PRP
					
					
[59] El Sharaki A. et al. (2023)	The comparison of Nd:YAG laser and PRP as an adjunct to SRP	30 patients with chronic periodontitis, patients’ mouths were divided into 2 sites. On one site, SRP + PRP was applied, on the other side, SRP + Nd:YAG	Clinical parameters (PD, GI, CAL, PI, and radiographic bony defects)	Baseline, 1 month, and 6 months post-treatment	The Nd:YAG laser is superior to PRP as it provides greater improvement of clinical parameters
					

The summary of included studies on PRP utility in non-surgical periodontal treatment, authors’ names, journals of publication, methodology, results assessment, and authors’ conclusions. PRP—platelet-rich plasma; SRP—scaling and root planing; PPD—periodontal probing depth; mBI—modified bleeding index; PI—plaque index; CAL—clinical attachment loss; PD—probing depth; GI—gingival index; BOP—bleeding on probing; i-PRF—injectable platelet-rich fibrin.

## Data Availability

No new data were created or analyzed in this study. Data sharing is not applicable to this article.

## Abbreviations

The following abbreviations are used in this manuscript:

PRP	Platelet-rich plasma
PRF	Platelet-rich fibrin
CGF	Concentrated growth factor
PPP	Platelet-poor plasma
SRP	Scaling and root planing
LPS	Lipopolysaccharides
EDTA	ethylene diamine tetraacetic acid
PDGF	Platelet-derived growth factor
TGF-β	Transforming growth factor
IGF-1	Insulin-like growth factor
i-PRF	Injectable PRF
L-PRF	Leukocyte platelet-rich fibrin
T-PRF	Titanium platelet-rich fibrin
A-PRF	Advanced platelet-rich fibrin
OHI	Oral hygiene index
GI	Gingival index
mBI	Modified bleeding index
PI	Plaque index
BoP	Bleeding on probing
PPD	Periodontal probing depth
CAL	Clinical attachment loss
PMPR	Professional mechanical plaque removal
Col-1	Collagen 1
LDD	Local drug delivery
GPCGF	Gel-phase CGF
LPCGF	Liquid-phase CGF
VEGF	Vascular endothelial growth factor
PG	Plasma gel
EGCG	Epigallocatechin-3-gallate
